# Patchwork: allele-specific copy number analysis of whole-genome sequenced tumor tissue

**DOI:** 10.1186/gb-2013-14-3-r24

**Published:** 2013-03-25

**Authors:** Markus Mayrhofer, Sebastian DiLorenzo, Anders Isaksson

**Affiliations:** 1 Science for Life Laboratory, Department of Medical Sciences, Uppsala University, SE-751 85 Uppsala, Sweden

**Keywords:** Cancer, allele-specific copy number analysis, whole-genome sequencing, aneuploidy, tumor heterogeneity, chromothripsis

## Abstract

Whole-genome sequencing of tumor tissue has the potential to provide comprehensive characterization of genomic alterations in tumor samples. We present Patchwork, a new bioinformatic tool for allele-specific copy number analysis using whole-genome sequencing data. Patchwork can be used to determine the copy number of homologous sequences throughout the genome, even in aneuploid samples with moderate sequence coverage and tumor cell content. No prior knowledge of average ploidy or tumor cell content is required. Patchwork is freely available as an R package, installable via R-Forge (http://patchwork.r-forge.r-project.org/).

## Background

Cancer is a disease in which somatic mutations lead to loss of proliferation control [1]. Genomic aberrations range from single-nucleotide mutations to copy number changes of sets of chromosomes, and can be recurrent in genomic regions, individual genes, and molecular pathways [2]. The number and complexity of genomic aberrations vary greatly between the different types of cancer. Recent large-scale studies have summarized the current knowledge in a genome-wide perspective [3-8].

Copy number aberrations affect both large and small portions of the genome. Methods such as spectral karyotyping (SKY) and comparative genome hybridization have provided progressively more detailed information on copy number aberrations [9-11]. With the introduction of high-density single-nucleotide polymorphism (SNP) arrays it is possible to obtain allele-specific information on a genome-wide scale [9,12]. Specialized software tools such as GAP (Genome Alteration Print), ASCAT (Allele-Specific Copy number Analysis of Tumors), and TAPS (Tumor Aberration Prediction Suite) were developed to use the allele-specific information to address issues such as aneuploidy and admixture of normal cells that complicate the analysis in tumor samples [13-15]. These tools provide allele-specific copy number analysis (ASCNA), that is, analysis of the absolute number of each homologous copy.

ASCNA can help identify the genotype of the amplified or deleted copy, which may have a direct implication on the tumor phenotype. Studies have shown that there may be preferential amplification of certain alleles in human tumors [16,17]. Perhaps more importantly, ASCNA helps interpret other somatic alterations, specifically point mutations. For example, if loss of heterozygosity (LOH) is detected in a region with a recessive mutation in a cancer-related gene, we can suspect a likely effect on tumor biology. ASCNA also facilitates reconstruction of the timing of mutational events through tumor development [2,18].

Recent advances in second-generation sequencing and data analysis are promoting whole-genome sequencing as an 'all-in-one' analysis for cancer genomes. Using whole-genome sequencing combined with bioinformatic tools it is possible to characterize an entire genome at base-pair resolution using a single molecular assay [19]. Several methods are available for copy number analysis of whole-genome sequencing data, but these do not provide absolute ASCNA [20,21]. Although tools that account for normal cell content have begun to emerge for whole-genome sequencing data [22], there is currently none that works without prior knowledge of the average ploidy. In this paper, we describe Patchwork, a tool for ASCNA of whole-genome sequencing data from tumor tissue. We found that performance was comparable with array-based methods in terms of resolution, sensitivity, and specificity, even with modest sequence coverage and thus this techniquie may obviate the need for copy number analysis based on SNP arrays.

## Results

ASCNA with Patchwork is based on the same principles as TAPS, which was developed for SNP array data [15]. Quantitative information on total and allele-specific DNA content is obtained for genomic segments, and visualized in relation to all segments in the genome. The observed pattern is used to estimate absolute copy numbers and purity, and to determine input parameters for automatic calling of allele-specific copy numbers.

**Figure 1:**
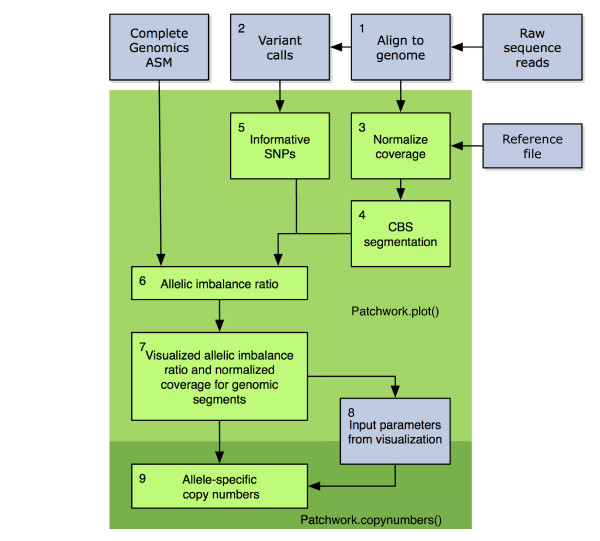
**Patchwork flowchart**. 1) Sequenced reads are aligned to the genome. 2) Single-nucleotide (and optionally short indel) variants that do not match the reference genome are extracted. 3) Systematic bias is removed by normalizing for GC content and other position-related effects. Coverage information from one or more diploid samples sequenced with the same method is used in this process. 4) The genome is segmented, based on the normalized coverage. 5) Informative heterozygous variants are identified. 6) Allelic imbalance ratio is calculated for each segment. 7) Visualization of allelic imbalance ratio and normalized coverage for genomic segments. 8) Manual interpretation of visualization to obtain input parameters for next step. 9) Allele-specific copy number is calculated for all genomic segments. Steps 3 to 7 and 9 are handled by the Patchwork.plot() and Patchwork.copynumbers() modules respectively.

Patchwork segments the genome based on total DNA content (normalized sequence coverage) using circular binary segmentation (CBS) [23]. For each segment, allele-specific information is used to estimate the relative abundance of the two homologous copies. Unless sequenced in great depth, it is unfeasible to obtain such an estimate from the allelic read counts of single SNPs. The actual coverage at a SNP is affected not only by copy number, but by sequence bias and random sampling, and therefore varies greatly from average coverage. However, along a segment containing many SNPs, a reliable measure of allelic imbalance can be achieved, even in samples with low coverage. In Patchwork, the allelic imbalance ratio of a genomic segment is calculated as

(∑high-∑low)/ ∑high,

where ∑low and ∑high are the number of reads with lower and higher observed allele counts summed over all heterozygous SNPs in the segment. Using sums of observed reads means that effectively each SNP is weighted according to its coverage, maximizing the use of the information. The allelic imbalance ratio and normalized coverage are plotted against each other for each genomic segment. Clusters of segments share allele-specific copy number. The allelic copy number compositions that emerge at different coverage levels can be used to discern the absolute copy numbers and, thereby, the average ploidy of the tumor cells. Figure 1 illustrates the steps undertaken by Patchwork to process input data into interpretable figures and allele-specific copy numbers. For further details, please see the Materials and methods section.

Patchwork can be used with any sequencing technology capable of producing SAM (Sequence alignment/map)-formatted aligned reads, which includes the most common sequencers from Roche, Illumina, and Life Technologies. In addition, ASM (assembly)-formatted data from Complete Genomics can be used directly in a version of Patchwork called PatchworkCG (Figure 1). A patient-matched normal sample or a reference file based on diploid samples sequenced with a similar technology is required. Reference files for Illumina/Solexa and Life Technologies/Solid are available with Patchwork. Users also have the option to build their own reference files from aligned reads obtained with their technology of choice.

### Performance validation using breast-cancer cell lines

We applied Patchwork ASCNA to high-coverage whole-genome sequencing data from the breast-cancer cell line HCC1187 (approx. 60× coverage; Complete Genomics Inc., Mountain View, CA, USA). Performance was evaluated using a TAPS analysis of HCC1187 (Affymetrix SNP 6.0; Affymetrix Inc., Santa Clara, CA, USA) as gold standard. Both datasets indicated an average ploidy of 2.6, and matched a SKY karyotype of HCC1187 [24]. Patchwork performance was evaluated using all Patchwork-generated segments of at least 1 Mb that overlapped by at least 75% with any TAPS segments (see Materials and methods for further details). The result of the performance evaluation is displayed in Table 1. Patchwork detected allele-specific copy numbers with a sensitivity of 93 to 100% and a specificity of 99 to 100%.

**Table 1:** Detection of allele-specific copy numbers in cell line HCC1187 using Patchwork, with (Affymetrix SNP 6

Total copies, n	Minor alleles, n	True positives, n	True negatives, n	False positives, n	False negatives, n	Sensitivity, %	Specificity, %
1	0	16	679	0	0	100.0	100.0
2	0	480	208	0	7	98.6	100.0
	1	7	688	0	0	100.0	100.0
3	0	39	649	6	1	97.5	99.1
	1	46	646	2	1	97.9	99.7
4	1	10	684	1	0	100.0	99.9
	2	69	618	3	5	93.2	99.5

^a^Number of segments, sensitivity and specificity are shown.

To evaluate the performance of Patchwork under more challenging conditions, we used whole-genome sequencing data from the breast-cancer cell line HCC1954 (approx. 4× coverage; Illumina GAII; Illumina Inc., San Diego, CA, USA) and patient-matched cell line HCC1954BL with normal karyotype (approx. 5× coverage; Illumina GAII). Sequencing reads were mixed from the two samples to resemble the effects of varying tumor cell content. A TAPS analysis of HCC1954 (Affymetrix SNP 6.0) was used as the gold standard. The estimated copy numbers closely resembled a publicly available SKY karyotype of HCC1954 [24], but there were some discrepancies between the sequencing, microarray, and SKY data. These discrepancies were whole chromosomes or chromosome arms that differed in copy number. The array, sequencing, and SKY data came from different sources and DNA extractions, and such differences can most likely be explained by gain or loss of chromosomes during culture. Cancer genomes are not necessarily stable during culture, and genomic alteration and subclones in the cell populations are frequently seen [15,18,25]. These chromosomes were excluded from the evaluation (see Materials and methods; see Additional file 1).

**Figure 2:**
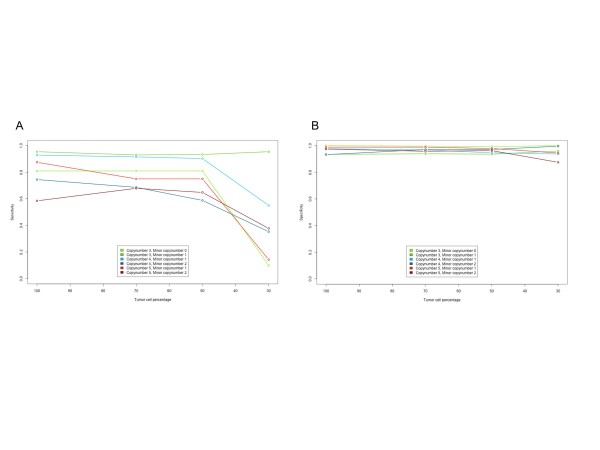
**Patchwork performance on *in silico *dilutions of cancer and matched normal cell lines**. TAPS (Tumor Aberration Prediction Suite) analysis of the breast-cancer cell line HCC1954 (nearly tetraploid) was used as a gold standard for the evaluation of Patchwork performance on a slightly different culture from the same cell line. Reads from the patient-matched blood cell line HCC1954BL were added to the data to estimate the effect of a reduced proportion of tumor cells. Data from the pure cancer cell line and mixtures corresponding to 70%, 50%, and 30% tumor cells were analyzed. **(A) **Sensitivity and **(B) **specificity for allele-specific copy number calls.

Sequence reads from HCC1954BL were used to dilute HCC1954 reads *in silico *into samples representing 70%, 50%, and 30% tumor cell content, and sensitivity and specificity were assessed for different allele-specific copy numbers (Figure 2A,B; for the numbers of matching segments, see Additional file 1). Some allele-specific copy numbers such as 3m1 and 4m1 were detected with both sensitivity and specificity above 92% for tumor proportions down to 50%; despite the low sequence coverage, this resembled performance with 60× coverage. For other copy numbers, performance was lower than expected. A closer examination of the Patchwork scatter plots indicated that short segments of relatively high copy number appeared outside the expected clusters. This may have been due to subclones in the cell population. Another explanation may be that 4× coverage of the cancer genome is just barely enough for Patchwork to separate these allele-specific copy numbers. Downsampling of HCC1954 to 2× coverage indicated additional performance loss on all copy numbers (data not shown).

### Patchwork analysis of a breast-cancer primary tumor and metastasis

**Figure 3:**
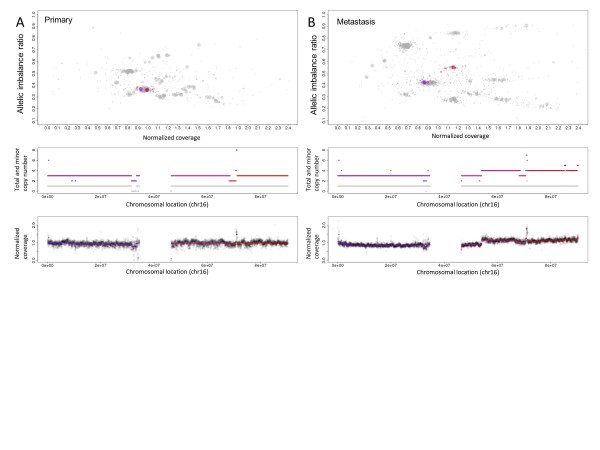
**Allele-specific copy number analysis of a breast-cancer primary tumor and metastasis**. **(A,B) **Patchwork analysis of **(A) **chromosome 16 of a breast-cancer primary tumor, and **(B) **of the same chromosome of a patient-matched metastasis. Top panels: Patchwork plot for genomic segments; middle panels: total and minor copy number for chromosome 16; bottom panel: normalized coverage for chromosome 16. With a few short deviations, the entire chromosome 16 displays heterozygous copy number 3 in the primary tumor sample. The copy number profile is nearly identical for the metastasis sample, except that most of the q-arm has yet another copy of the amplified chromosome.

We applied Patchwork to whole-genome sequencing data from a breast-cancer primary tumor, metastasis and xenograft (approx. 30× coverage; Illumina GAII) from a published study [26]; results of Patchwork visualizations for the primary tumor and the metastasis are shown in Figure 3. In the top panel, segments from the entire genome seem to be closer to each other in the primary tumor (Figure 3A), which indicates a lower proportion of tumor cells than in the metastasis sample (Figure 3B). A description of how normalized coverage is affected by the proportion of tumor cells can be found in the Materials and methods section.

We found that 97% of the genome (base pairs) matched in terms of total copy gain, total copy loss, and unchanged copy number between the primary tumor and metastasis, indicating a high similarity (see Additional file 2). The average ploidy was almost 3.5 for both samples, and allele-specific copy numbers were mostly identical throughout the genome. One exception was that most of chromosome 16q had three copies in the primary tumor and four copies in the metastasis, with retained heterozygosity in both samples (Figure 3AB). The xenograft displayed very variable sequence coverage, likely due to contamination by mouse DNA. We used the Patchwork figures to visually compare the samples, and found no copy number differences between the xenograft and the primary tumor. This is further illustrated in whole-genome copy number profiles in Additional file 2.

Our analysis indicated systematically higher copy numbers than the originally published copy number analysis by Ding *et al. *[26], which may be due to the original analysis not taking the true average ploidy (approx. 3.5) into account, and thus underestimating the copy numbers. In addition, the findings by Ding *et al*. of more copy number alterations in the metastasis may be due to a lower sensitivity of detection in the primary tumor, which seems to have lower tumor cell content (Figure 3A,B; see Additional file 2).

### A detailed view of chromothripsis

**Figure 4:**
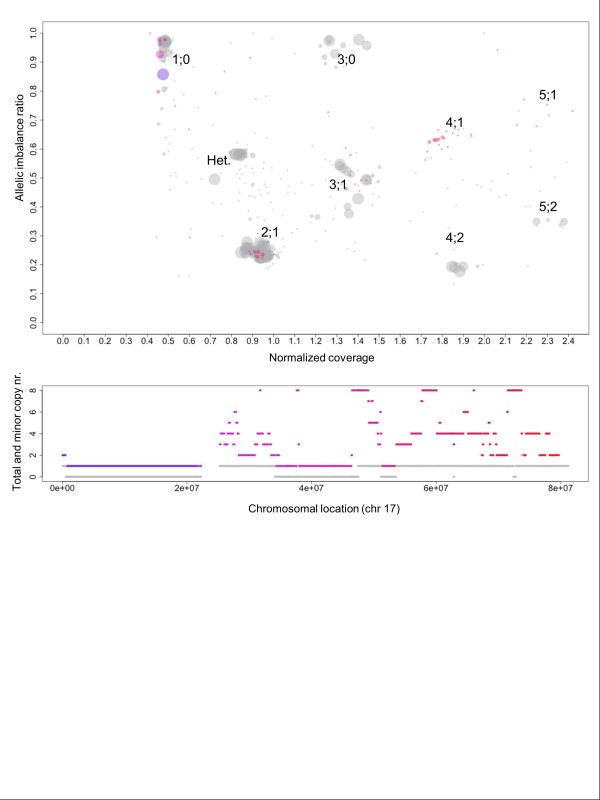
**Detailed visualization of chromothripsis in a breast-cancer cell line**. On chromosome 17 of the breast-cancer cell line HCC2218, total copy number varies on the q-arm whereas the minor copy number stays at 1. This is consistent with one of the homologs having undergone chromothripsis. Top panel: Scatter plot of allelic imbalance ratio and normalized coverage for segments on chromosome 17, colored in blue-red gradient. Grey segments are those located on other chromosomes. Bottom panel: Total copy number in blue-red gradient depending on chromosomal location. Grey indicates minor copy number.

Chromothripsis is a catastrophic rearrangement of chromosomal regions that includes deletions and amplifications [27]. It has been associated with formation of fusion genes and double minute chromosomes. Typically, only one of the two homologous chromosomes is affected by the phenomenon, leaving the other(s) intact. We used Patchwork to analyze data from the breast-cancer cell line HCC2218 (approx. 60× coverage; Complete Genomics) and found an example of chromothripsis on chromosome 17. All segments of chromosome 17 are shown in Figure 4. On a large portion of the q-arm, the highly variable normalized coverage indicates non-integer total copy numbers. ASCNA (bottom panel) indicates that the minor copy number remains at 1. This pattern matches those described for chromothripsis, and is consistent with the theory of fragmentation of one of the homologs, followed by aberrant multiplication, loss, and reassembly of the fragments [27]. Genes in the region may have been damaged, lost or amplified, coupled with a different promoter, or fused with another gene. Analysis of copy numbers (which may be heterogeneous and therefore appear non-integer) is an important part of understanding the cancer genome in these highly aberrant regions.

## Discussion

### The importance of copy number analysis

Characterization of cancer genomes benefit from ASCNA in three major ways. 1) ASCNA provides an accurate measure of total copy number in cases of aneuploidy. Finding the correct copy numbers rather than calling gain or loss relative to the average coverage, as is commonly done, may avoid false discovery of homozygous loss. 2) ASCNA reveals LOH, which indicates whether the tumor cells retain a constitutive allele that may render recessive mutations inconsequential. 3) ASCNA facilitates the identification and analysis of shattered chromosomes (chromothripsis), which is being recognized as an important type of genomic aberration and may be associated with poor prognosis.

We believe that allele-specific copy numbers and normal cell content should be a part of the input information for analysis of events such as translocation breakpoints, point mutations, and short insertions and deletions. Because allele-specific copy numbers reflect the composition of the homologous copies along the genome, they can be used to reconstruct the set of events leading to formation of the observed cancer genome [18]. Restricting the analysis to total copy number and LOH may limit our understanding of the molecular genetic events that have taken place in a cancer genome.

### Limitations

Patchwork provides information on total copy number and the number of copies of the minor allele, but does not assign copy number to specific SNP alleles, which may be desired, as certain alleles may be preferentially amplified in some tumors. Specialized methods are available for this purpose, joining copy number and genotyping analysis. One such tool is HATS (Haplotype Amplification in Tumor Sequences), developed for identification of amplified alleles using haplotype information [28]. We suggest using Patchwork to identify allele-specific copy numbers, and HATS to identify individual higher-copy SNP alleles in regions where the original homologs have unequal copy number. It should be pointed out that HATS is not designed to identify which haplotype is the background for somatic mutations. Reads covering both the somatic mutation and a second polymorphic site can be used for that purpose [29].

Patchwork is designed for whole-genome sequencing data. Although most aspects of Patchwork would also be viable for whole exome sequencing, such data are different in some important respects. Exome sequencing relies on enrichment strategies that may cause saturation effects and require different normalization. Sequencing of such a small portion of the genome reduces the number of informative heterozygous markers and requires a different segmentation solution. Other tools have been developed specifically for detection of copy number aberrations and LOH in cancer from exome sequencing data [30,31]; however, they do not provide ASCNA nor take aneuploidy into account.

Copy number analysis is still usually performed using SNP arrays because of the lower costs and DNA requirements, easier data handling, and mature analysis tools. With Patchwork, we have taken an analysis strategy originally conceived for SNP arrays, and transformed it into a tool that extracts similar data from whole-genome sequencing. After normalization and SNP identification, the analysis strongly resembles that of array data. Within the sample, a relative change in signal intensity (normalized for sequence or hybridization bias) represents a change in copy number. Whereas microarrays are subject to hybridization effects such as saturation (limiting sensitivity at high copy numbers), normalized sequence-read coverage is proportional to the copy number of the original cells. Another potential advantage with sequencing is that paired-end assays and/or local reassembly of reads can be used to map breakpoints in greater detail than with microarrays. We expect that future versions of Patchwork will be able to use such information to complement CBS and generate a much more detailed characterization of the cancer genome than is currently possible with SNP arrays.

### The Patchwork software

The Patchwork website (http://patchwork.r-forge.r-project.org/) has documentation and links to available R packages, installable via R-forge [32]. Currently two versions are available, one for use on BAM (Binary sequence alignment/map)-formatted data and one for use on ASM-formatted (Complete Genomics) data. Detailed instructions, including examples and tutorials are also available. Patchwork runs on desktop computers.

## Conclusions

Many studies have shown that analysis of copy numbers and LOH is an important part of genome characterization in cancer, and that DNA microarrays are suitable for the task. Bioinformatic tools capable of ASCNA of cancer genomes have been available for SNP array data for some time, but tools for whole-genome sequencing data have lagged behind. With Patchwork, we have developed a tool with which whole-genome sequencing, even at modest sequence coverage, can be used for ASCNA of cancer genomes.

## Materials and methods

### Patchwork data input

Patchwork takes BAM-formatted aligned reads as input, which is the standard output from most short-read aligners. ASM-formatted data (Complete Genomics) are also supported, and other formats may be added in the near future. Single-nucleotide variant data (for allele-specific quantification) is extracted using SAMtools [33], and discovered variants are filtered using a list of known SNPs (dbSNP) [34]. If a patient-matched normal sample is available, it is used to improve the ability of Patchwork to identify constitutive heterozygous SNPs, which are informative for allele-specific analysis.

### GC content normalization

BAM formatted data are divided into short (200 bp) windows, which are normalized for GC content bias. The normalization process groups the windows based on GC content (extracted from, in this case, the human genome assembly hg19) and normalizes each group based on the read count of each window relative to the group average. This strategy resembles what is used in other methods and is extremely effective because GC content tends to correlate non-linearly with sequence coverage, and differs depending on the sequencing platform and library preparation [22].

### Positional normalization

For normalization of unknown positional bias, Patchwork uses either a patient-matched normal sample or a reference file based on diploid samples sequenced with the same sequencing protocol. The reference data are normalized for GC content as described above, and in case of several reference samples, averaged for each 200 bp window. Reference files are provided for Illumina/Solexa and Life Technologies/Solid data, and can easily be prepared for other types of data.

### Smoothing and segmentation

Normalized coverage, relative to the reference, is summarized in 10 kb, fixed-window bins along each chromosome arm. Chromosome arms are then individually segmented using circular binary segmentation (the DNAcopy package) [35]. Each segment is assigned average normalized coverage and allelic imbalance ratio, which is

(∑high-∑low)/ ∑high,

where ∑low and ∑high are the number of reads with lower and higher observed allele counts, summed for all heterozygous SNPs in the segment.

### Copy number visualization and analysis

Patchwork generates color-coded figures for each chromosome, with a gradient from blue on the distal p-arm to red on the distal q-arm. These figures form the primary result, and allow the analyst to interpret the sample in terms of average ploidy, coverage and copy number relationship, LOH, tumor cell content, and tumor cell heterogeneity. Ploidy can be determined from the cluster pattern, with one possible cluster for copy number 1 (1m0), two possible clusters for copy number 2 (2m1 and 2m0), and so on. An automated copy number calling method similar to that of TAPS is also available. It requires an initial interpretation of the figures (currently the approximate coverage difference of a single copy, and the allelic imbalance ratios corresponding to copy number 2 with and without LOH). The algorithm assigns allele-specific copy number to genomic segments, based on the initial interpretation and knowledge of the figure patterns.

The copy number analysis algorithm also calculates the average ploidy and purity of the tumor cells. The average ploidy, Ploidy_Tum_, is the average total copy number of all genomic segments weighted by segment length. The purity estimate is based on Δ_obs_, the observed difference in normalized coverage, corresponding to one copy in the tumor cells, and Δ_exp_, which is the difference that can be expected in a sample with 100% pure tumor cells. Δ_exp _is obtained from Ploidy_Tum _(equation 1). For example, a triploid sample has three copies at the average normalized coverage, and loss or gain of one copy in a particular region would be expected to alter the normalized coverage by one-third. Copy number alterations in a fraction of the DNA would further reduce the effect by that fraction. Thus, the tumor DNA content DNAfrac_Tum _can be expressed in terms of Δ_obs _and Δ_exp_, (equation 2). DNAfrac_Tum _is also a function of tumor cell content (purity) Cellfrac_Tum _and the ploidy of the tumor and normal cells Ploidy_Tum _and Ploidy_Norm _(equation 3). Patchwork assumes that Ploidy_Norm _= 2 and Cellfrac_Norm _= 1-Cellfrac_Tum _and calculates Cellfrac_Tum _accordingly (equation 4).

(1) $$\Delta_{\text{exp}}=\frac{1}{\text{Ploid}{\text{y}}_{\text{Tum}}}$$

(2) $$\text{DNAfra}{\text{c}}_{\text{Tum}}=\frac{\Delta_{\text{obs}}}{\Delta_{\text{exp}}}$$

(3) $$\text{DNAfra}{\text{c}}_{\text{Tum}}=\frac{\text{Cellfra}{\text{c}}_{\text{Tum}}*\text{Ploid}{\text{y}}_{\text{Tum}}}{\text{Cellfra}{\text{c}}_{\text{Tum}}*\text{Ploid}{\text{y}}_{\text{Tum}}+\text{Cellfra}{\text{c}}_{\text{Norm}}*\text{Ploid}{\text{y}}_{\text{Norm}}}$$

(4) $$\text{Cellfra}{\text{c}}_{\text{Tum}}=\frac{1}{1+\frac{\text{Ploid}{\text{y}}_{\text{Tum}}}{2}\left(\frac{1}{\text{DNAfra}{\text{c}}_{\text{Tum}}}-1\right)}$$

### Data acquisition and processing

Microarray data (Affymetrix SNP6) for HCC1954 and HCC1187 were acquired from GEO [GEO:GSE13372; GSE36138], preprocessed in Nexus Copy Number (version 5.0) and analyzed for allele-specific copy number using TAPS. SKY karyotypes of HCC1187 and HCC1954 were acquired from the University of Cambridge [24].

Sequence data from HCC1954/HCC1954BL originally published by Chiang *et al. *[21], was obtained from SRA [SRA:SRA001246] and aligned to the 'hg19' human genome assembly from UCSC using Bowtie [36]. Sequenced reads from a breast-cancer primary tumor, matched non-tumor tissue, metastasis and xenograft, originally published by Ding *et al. *[26] were obtained from dbGAP [phs000245.v1.p1] and aligned using Bowtie. ASM-formatted sequence data from HCC1187 and HCC2218 (assembly software version 2.0.0.32) were obtained from Complete Genomics [37,38].

### Performance evaluations

The sequenced reads from the HCC1954 cancer cell line were diluted by adding reads from the patient-matched blood cell line HCC1954BL using a random-number generator. Reads were selected with a probability based on HCC1954 total coverage, HCC1954 average ploidy (nearly tetraploid), and the desired tumor cell content. The diluted samples and the ASM-formatted sequence data from Complete Genomics were analyzed with Patchwork.

For HCC1187 and HCC1954, sensitivity and specificity were calculated for each allele-specific copy number by comparing the Patchwork results, with the TAPS (microarray) gold standard. Patchwork-generated segments larger than 1 Mb with at least 75% overlap with the microarray data were used. Exact total and minor copy number matching was required. Sensitivity was calculated as true positives/(true positives + false negatives) and specificity as true negatives/(true negatives + false positives). Chromosomes 5, 8, 13, 15, and 17 were excluded from the analysis of the HCC1954 cell line (see Results section; see Additional file 1 Supplemental data). Performance results for the most abundant copy number compositions (>15 segments) were used for Figure 2. The accuracy of the TAPS analysis was confirmed using publicly available SKY karyotypes [24].

### Breast-cancer tissue samples

Similarity of Patchwork results from the breast-cancer primary tumor and metastasis samples was confirmed by matching average copy number and gain, loss, or unchanged copy number along the genome at base-pair resolution.

## Abbreviations

ASCAT: Allele-Specific Copy number Analysis of Tumors; ASCNA: Allele-specific copy number analysis; BAM: Binary sequence alignment/map; CBS: Circular binary segmentation; GAP: Genome Alteration Print; dbGAP: Database of Genotypes and Phenotypes; GEO: Gene Expression Omnibus; HATS: Haplotype Amplification in Tumor Sequences; LOH: Loss of heterozygosity; SAM: Sequence alignment/map; SKY: Spectral karyotyping; SNP: Single-nucleotide polymorphism; SRA: Sequence Read Archive; TAPS: Tumor Aberration Prediction Suite

## Competing interests

The authors declare that they have no competing interests.

## Authors' contributions

MM designed and implemented the method, performed analyses, and wrote the paper. SD implemented the method, wrote documentation, and performed analyses. AI planned method development and study design, and wrote the paper. All authors read and approved the final manuscript.

## Breast-cancer tissue dataset

Funding support for the breast-cancer primary tumor, metastasis and xenograft sequence data was provided by grants from Washington University in St. Louis and the National Human Genome Research Institute (NHGRI U54 HG003079), the National Cancer Institute (NCI 1 U01 CA114722-01), the Susan G Komen Breast Cancer Foundation (BCTR0707808), and the Fashion Footwear Charitable Foundation, Inc. NCI U10 CA076001. Breast Cancer Research Foundation grant awarded to the American College of Surgeons Oncology Group supported the acquisition of samples for recurrence testing. The tissue procurement core was supported by an NCI core grant (NCI 3P50 CA68438). The Human and Mouse Linked Evaluation of Tumors Core was supported by the Institute of Clinical and Translational Sciences at Washington University (CTSA grant UL1 RR024992). Illumina, Inc. and Washington University also supported this dataset through the Washington University Cancer Genome Initiative.

## Supplementary Material

Additional File 1 **Comparison of TAPS (Tumor Aberration Prediction Suite) and Patchwork analyses of the breast-cancer cell line HCC1954**. Click here for file

Additional File 2 Patchwork copy number profiles of breast-cancer primary tumor, metastasis, and xenograft, based on sequence data originally published by Ding *et al. *[26]. Click here for file
