# Supplementary material for: Patchwork: allele-specific copy number analysis of whole-genome sequenced tumor tissue
Source: Genome Biol. 2013 Mar 25;14(3):R24. doi: 10.1186/gb-2013-14-3-r24 (PMC4053982; doi:10.1186/gb-2013-14-3-r24)
Supplement: Additional File 1 — Comparison of TAPS (Tumor Aberration Prediction Suite) and Patchwork analyses of the breast-cancer cell line HCC1954. [file gb-2013-14-3-r24-S1.PPT]

## Slide 1
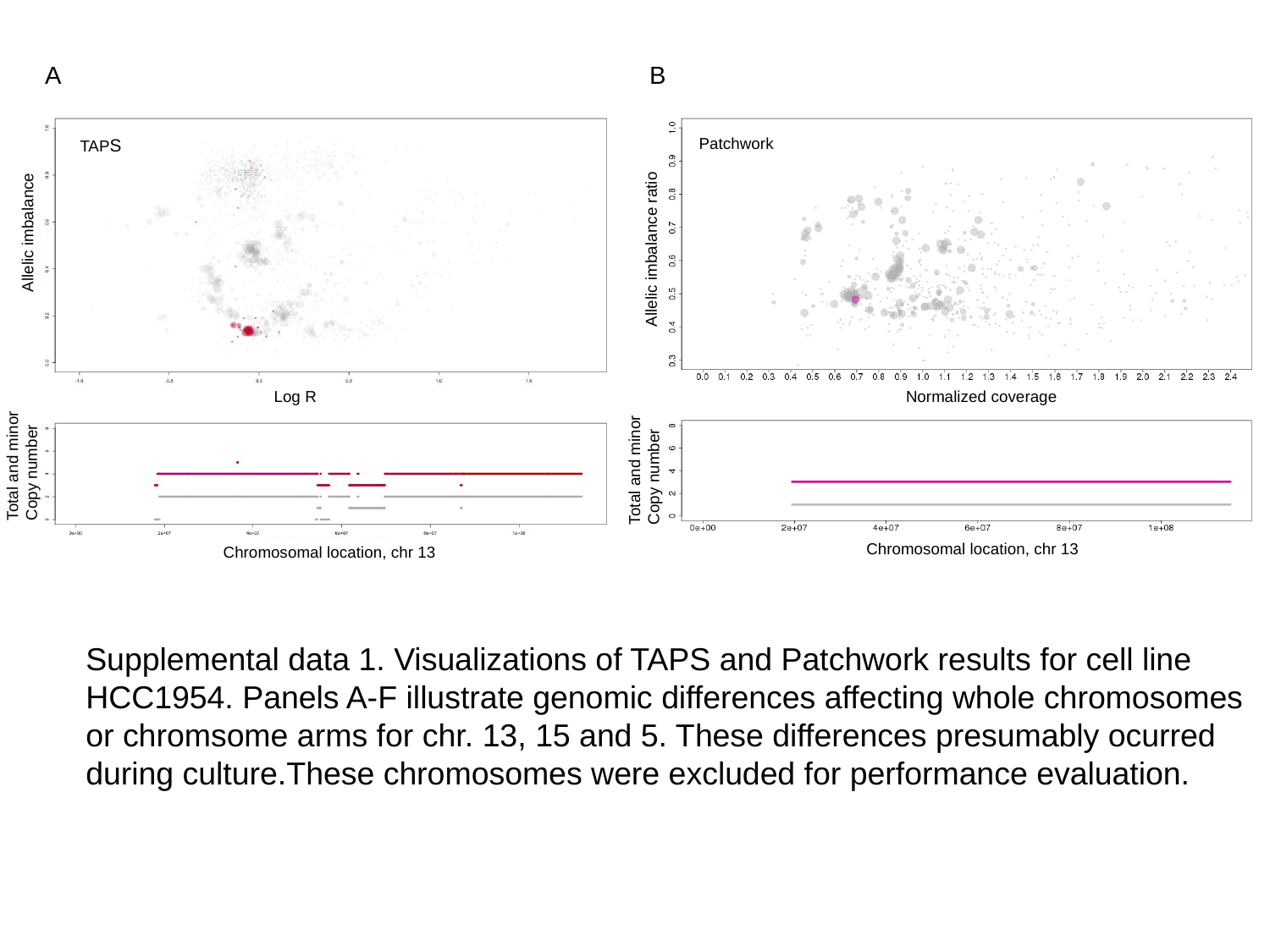

A
B
Patchwork
TAPS
Allelic imbalance
Allelic imbalance ratio
Log R
Normalized coverage
Total and minor
Copy number
Total and minor
Copy number
Chromosomal location, chr 13
Chromosomal location, chr 13
Supplemental data 1. Visualizations of TAPS and Patchwork results for cell line
HCC1954. Panels A-F illustrate genomic differences affecting whole chromosomes
or chromsome arms for chr. 13, 15 and 5. These differences presumably ocurred
during culture.These chromosomes were excluded for performance evaluation.

## Slide 2
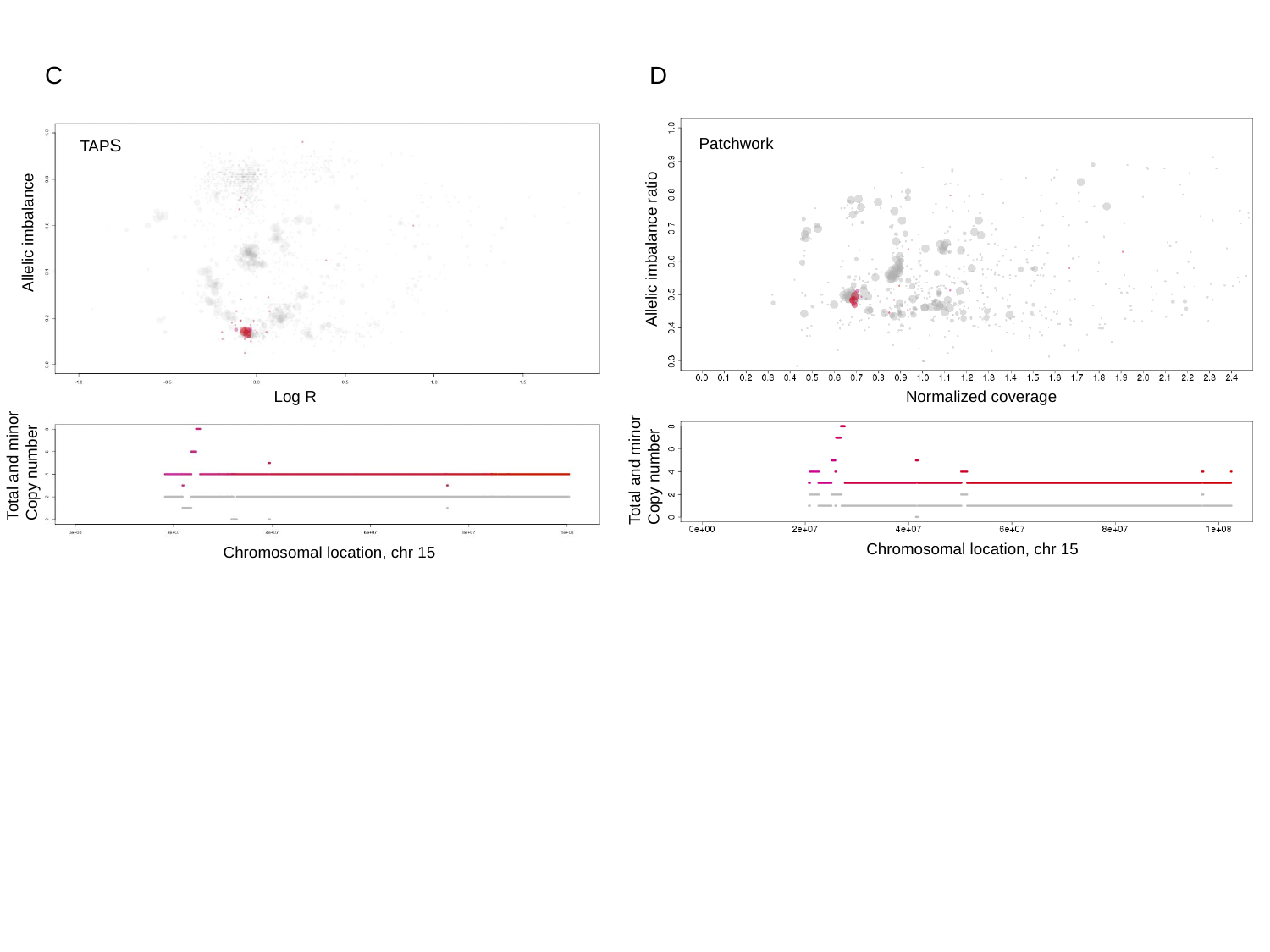

C
D
Patchwork
TAPS
Allelic imbalance
Allelic imbalance ratio
Log R
Normalized coverage
Total and minor
Copy number
Total and minor
Copy number
Chromosomal location, chr 15
Chromosomal location, chr 15

## Slide 3
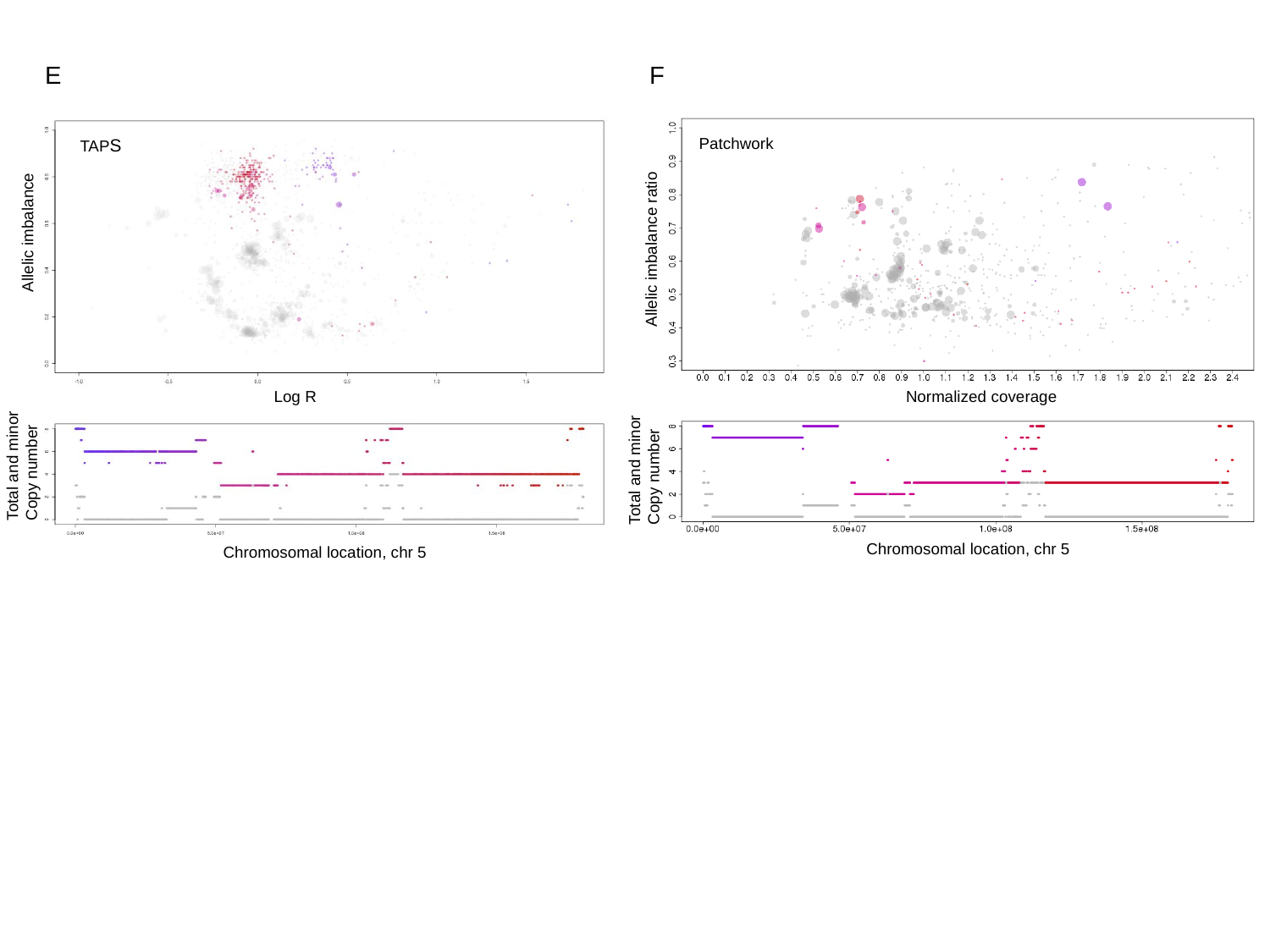

E
F
Patchwork
TAPS
Allelic imbalance
Allelic imbalance ratio
Log R
Normalized coverage
Total and minor
Copy number
Total and minor
Copy number
Chromosomal location, chr 5
Chromosomal location, chr 5

## Slide 4
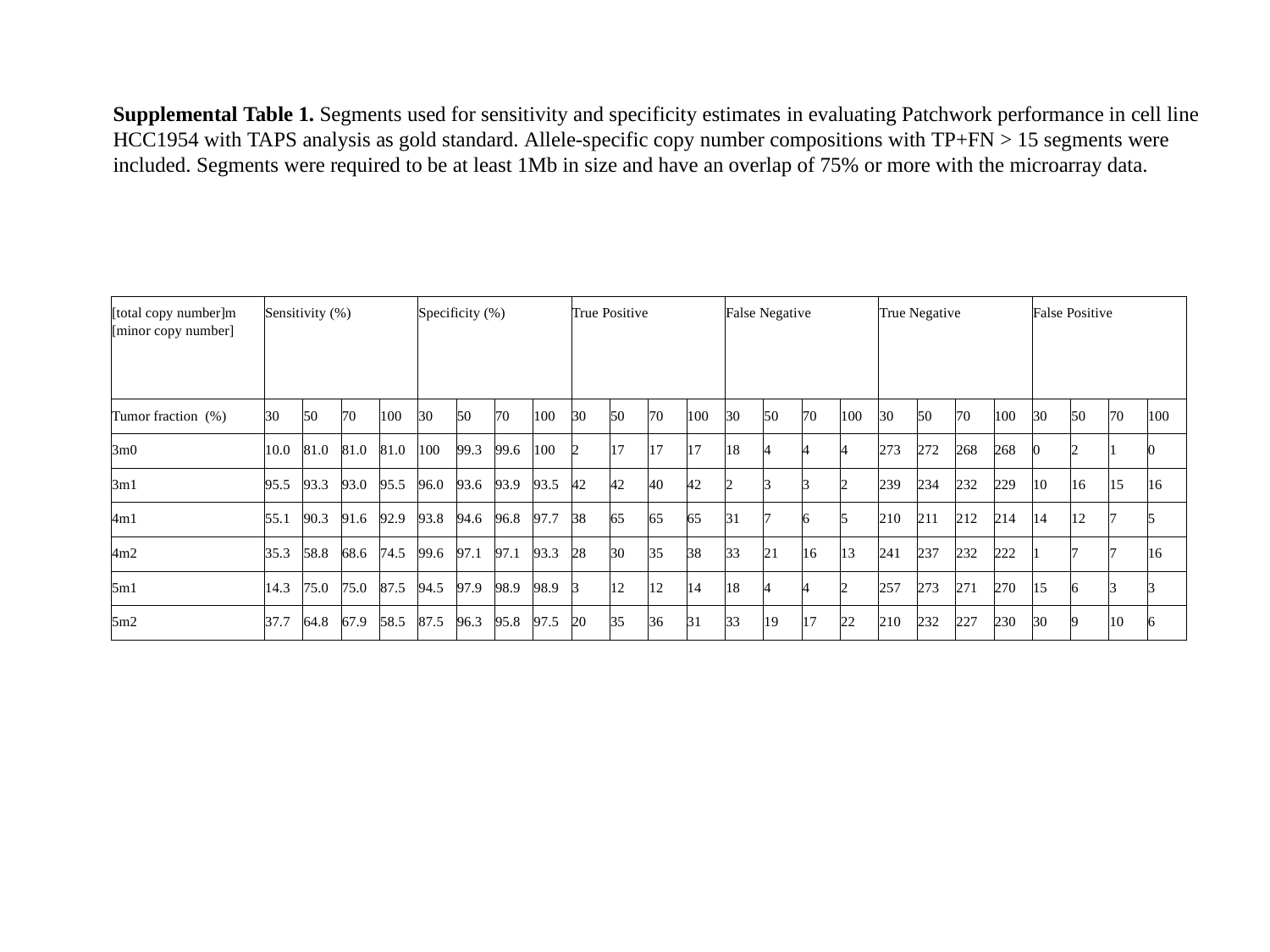

Supplemental Table 1. Segments used for sensitivity and specificity estimates in evaluating Patchwork performance in cell line HCC1954 with TAPS analysis as gold standard. Allele-specific copy number compositions with TP+FN > 15 segments were included. Segments were required to be at least 1Mb in size and have an overlap of 75% or more with the microarray data.
| [total copy number]m [minor copy number] | Sensitivity (%) | | | | Specificity (%) | | | | True Positive | | | | False Negative | | | | True Negative | | | | False Positive | | | |
| --- | --- | --- | --- | --- | --- | --- | --- | --- | --- | --- | --- | --- | --- | --- | --- | --- | --- | --- | --- | --- | --- | --- | --- | --- |
| Tumor fraction (%) | 30 | 50 | 70 | 100 | 30 | 50 | 70 | 100 | 30 | 50 | 70 | 100 | 30 | 50 | 70 | 100 | 30 | 50 | 70 | 100 | 30 | 50 | 70 | 100 |
| 3m0 | 10.0 | 81.0 | 81.0 | 81.0 | 100 | 99.3 | 99.6 | 100 | 2 | 17 | 17 | 17 | 18 | 4 | 4 | 4 | 273 | 272 | 268 | 268 | 0 | 2 | 1 | 0 |
| 3m1 | 95.5 | 93.3 | 93.0 | 95.5 | 96.0 | 93.6 | 93.9 | 93.5 | 42 | 42 | 40 | 42 | 2 | 3 | 3 | 2 | 239 | 234 | 232 | 229 | 10 | 16 | 15 | 16 |
| 4m1 | 55.1 | 90.3 | 91.6 | 92.9 | 93.8 | 94.6 | 96.8 | 97.7 | 38 | 65 | 65 | 65 | 31 | 7 | 6 | 5 | 210 | 211 | 212 | 214 | 14 | 12 | 7 | 5 |
| 4m2 | 35.3 | 58.8 | 68.6 | 74.5 | 99.6 | 97.1 | 97.1 | 93.3 | 28 | 30 | 35 | 38 | 33 | 21 | 16 | 13 | 241 | 237 | 232 | 222 | 1 | 7 | 7 | 16 |
| 5m1 | 14.3 | 75.0 | 75.0 | 87.5 | 94.5 | 97.9 | 98.9 | 98.9 | 3 | 12 | 12 | 14 | 18 | 4 | 4 | 2 | 257 | 273 | 271 | 270 | 15 | 6 | 3 | 3 |
| 5m2 | 37.7 | 64.8 | 67.9 | 58.5 | 87.5 | 96.3 | 95.8 | 97.5 | 20 | 35 | 36 | 31 | 33 | 19 | 17 | 22 | 210 | 232 | 227 | 230 | 30 | 9 | 10 | 6 |
