# Supplementary material for: Patchwork: allele-specific copy number analysis of whole-genome sequenced tumor tissue
Source: Genome Biol. 2013 Mar 25;14(3):R24. doi: 10.1186/gb-2013-14-3-r24 (PMC4053982; doi:10.1186/gb-2013-14-3-r24)
Supplement: Additional File 2 — Patchwork copy number profiles of breast-cancer primary tumor, metastasis, and xenograft, based on sequence data originally published by Ding et al. [26]. [file gb-2013-14-3-r24-S2.PPT]

## Slide 1
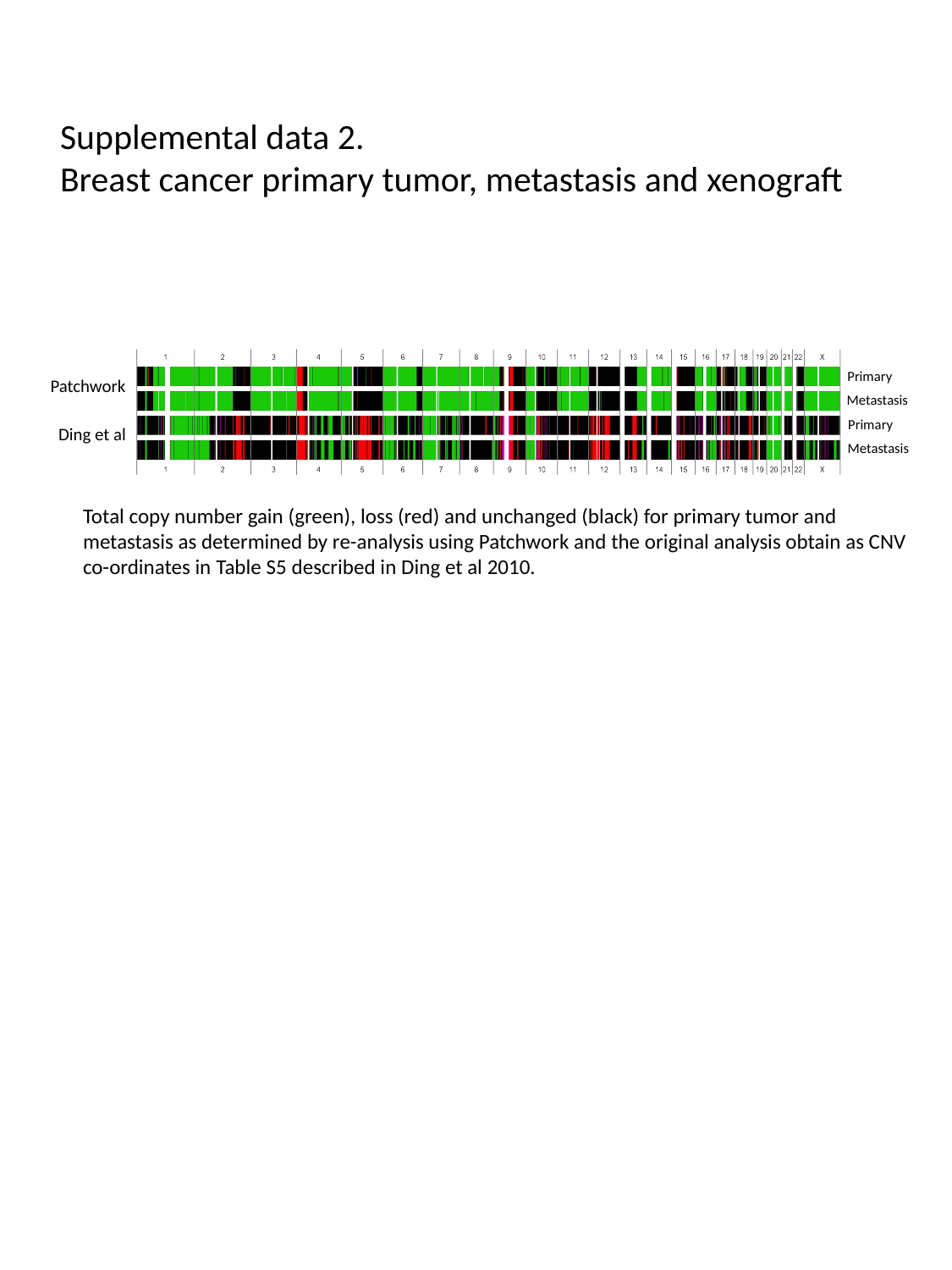

# Supplemental data 2. Breast cancer primary tumor, metastasis and xenograft
Primary
Patchwork
Metastasis
Primary
Ding et al
Metastasis
Total copy number gain (green), loss (red) and unchanged (black) for primary tumor and metastasis as determined by re-analysis using Patchwork and the original analysis obtain as CNV co-ordinates in Table S5 described in Ding et al 2010.

## Slide 2
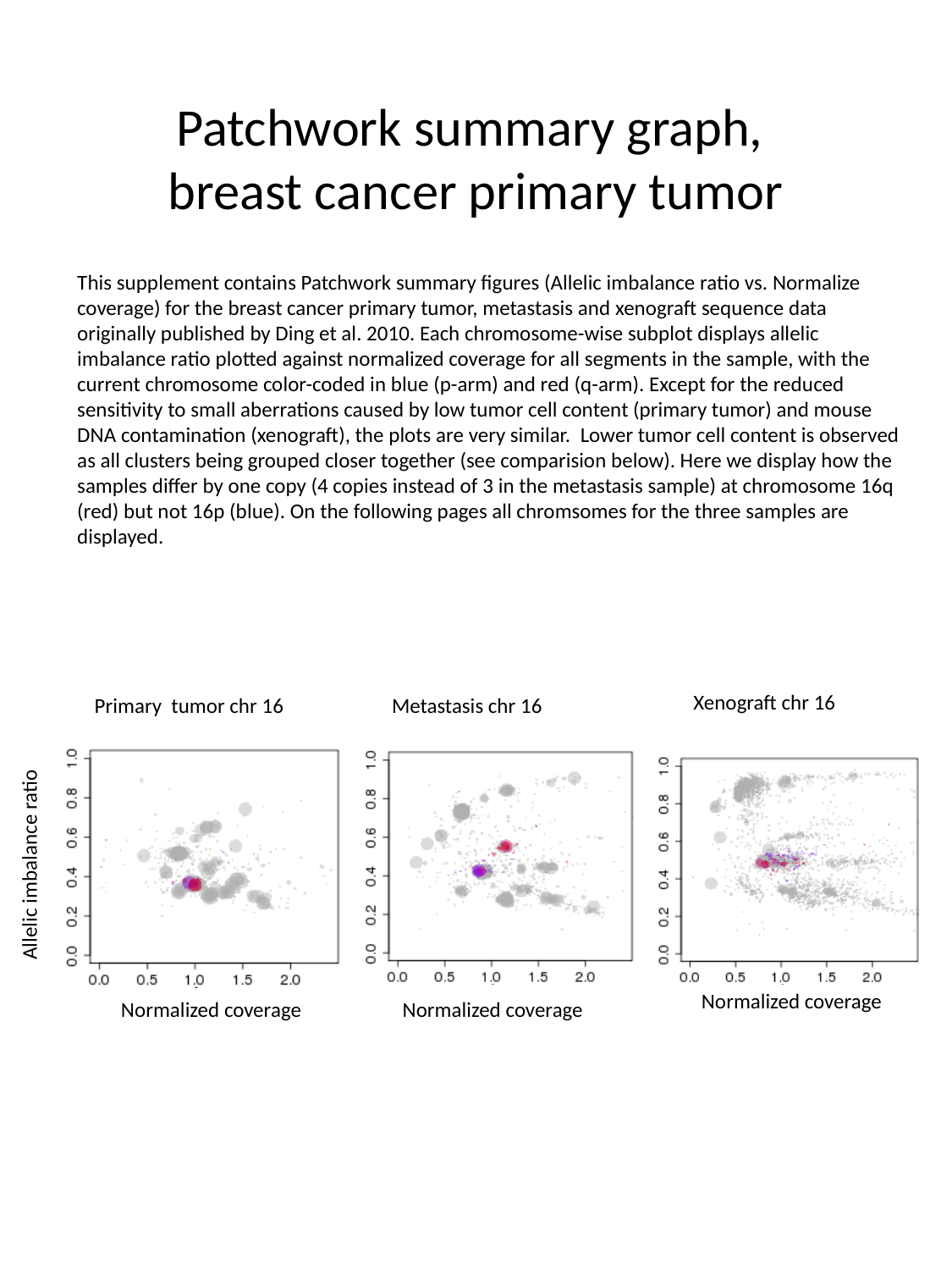

# Patchwork summary graph, breast cancer primary tumor
This supplement contains Patchwork summary figures (Allelic imbalance ratio vs. Normalize coverage) for the breast cancer primary tumor, metastasis and xenograft sequence data originally published by Ding et al. 2010. Each chromosome-wise subplot displays allelic imbalance ratio plotted against normalized coverage for all segments in the sample, with the current chromosome color-coded in blue (p-arm) and red (q-arm). Except for the reduced sensitivity to small aberrations caused by low tumor cell content (primary tumor) and mouse DNA contamination (xenograft), the plots are very similar. Lower tumor cell content is observed as all clusters being grouped closer together (see comparision below). Here we display how the samples differ by one copy (4 copies instead of 3 in the metastasis sample) at chromosome 16q (red) but not 16p (blue). On the following pages all chromsomes for the three samples are displayed.
Xenograft chr 16
Primary tumor chr 16
Metastasis chr 16
Allelic imbalance ratio
Normalized coverage
Normalized coverage
Normalized coverage

## Slide 3
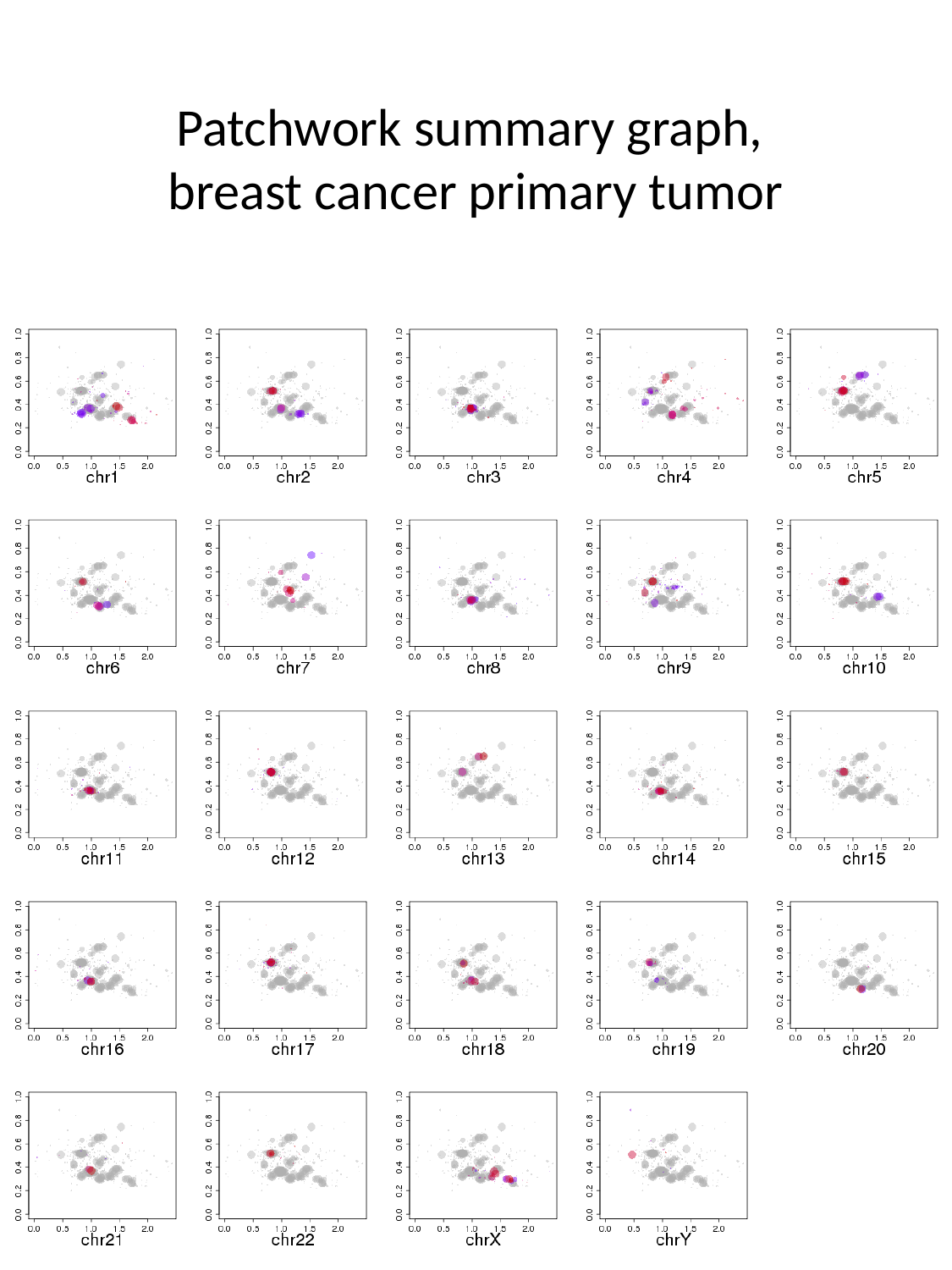

# Patchwork summary graph, breast cancer primary tumor

## Slide 4
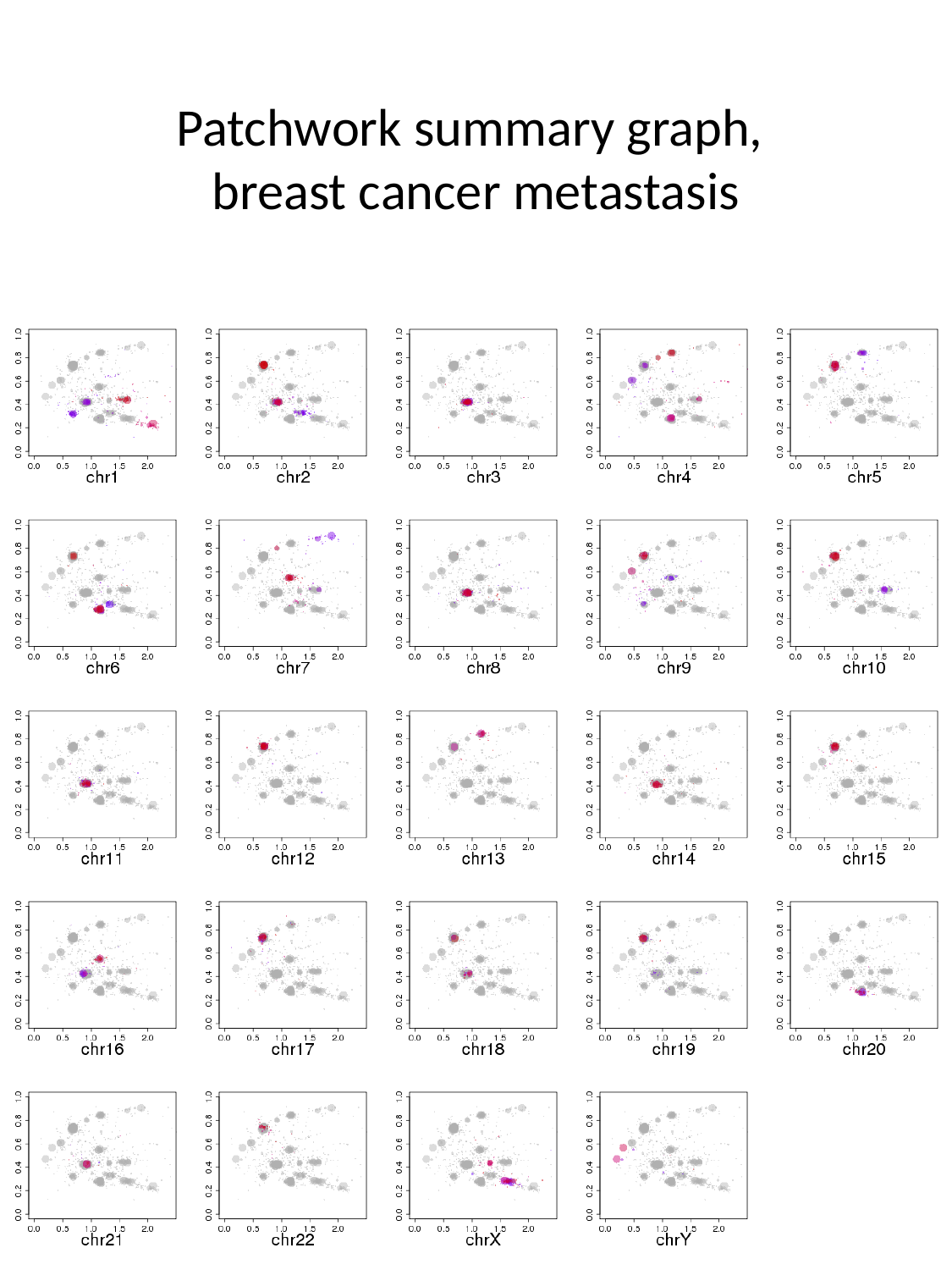

# Patchwork summary graph, breast cancer metastasis

## Slide 5
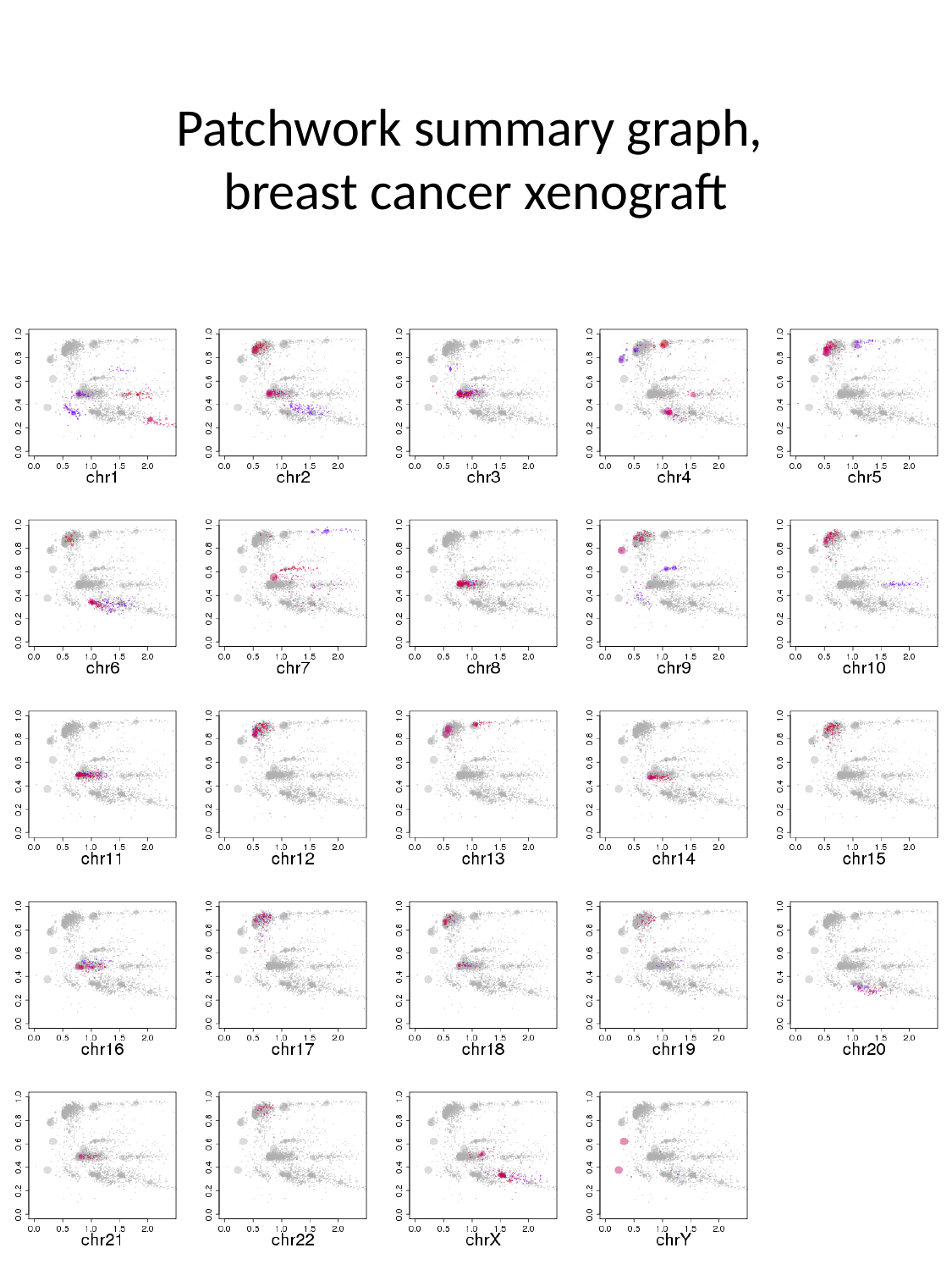

# Patchwork summary graph, breast cancer xenograft
